# Fyn Signaling Is Compartmentalized to Dopamine D1 Receptor Expressing Neurons in the Dorsal Medial Striatum

**DOI:** 10.3389/fnmol.2017.00273

**Published:** 2017-08-31

**Authors:** Khanhky Phamluong, Emmanuel Darcq, Su Wu, Samuel A. Sakhai, Dorit Ron

**Affiliations:** Department of Neurology, University of California San Francisco San Francisco, CA, United States

**Keywords:** Fyn, NMDA, striatum, dopamine, signal transduction, RACK1, scaffolding proteins, lipid rafts

## Abstract

The tyrosine kinase Fyn plays an important role in synaptic plasticity, learning, and memory. Here we report that Fyn is activated in response to 15 min D1 receptor (D1R) but not D2 receptor (D2R) stimulation specifically in the dorsomedial striatum (DMS) of mice but not in the other substriatal regions, the dorsolateral striatum (DLS), and the nucleus accumbens (NAc). Once activated Fyn phosphorylates its substrate GluN2B, and we show that GluN2B is phosphorylated only in the DMS but not in the other striatal regions. Striatal neurons are divided into D1R expressing medium spiny neurons (MSNs) and D2R expressing MSNs. Thus, to explore the cell-type specificity of this signaling pathway in the DMS, we developed a Cre-dependent Flip Excision (FLEX) approach to knockdown Fyn in D1R MSNs or D2R MSNs, and proved that the D1R-dependent Fyn activation is localized to DMS D1R MSNs. Importantly, we provide evidence to suggest that the differential association of Fyn and GluN2B with the scaffolding RACK1 is due to the differential localization of Fyn in lipid rafts.Our data further suggest that the differential cholesterol content in the three striatal regions may determine the region specificity of this signaling pathway. Together, our data show that the D1R-dependent Fyn/GluN2B pathway is selectively activated in D1R expressing MSNs in the DMS, and that the brain region specificity of pathway depends on the molecular and cellular compartmentalization of Fyn and GluN2B.

## Introduction

Fyn kinase belongs to the Src family of protein tyrosine kinases (PTKs) (Resh, 1998). Fyn is highly expressed throughout the developing and adult brain (Umemori et al., 1992; Yagi et al., 1993). Fyn plays an important role in excitatory and inhibitory synaptic transmission (Ohnishi et al., 2011; Trepanier et al., 2012; Chattopadhyaya et al., 2013; Hildebrand et al., 2016), synaptic plasticity learning and memory (Grant et al., 1992; Kojima et al., 1997; Salter and Kalia, 2004). In addition, Fyn also participates in pathologies such as alcohol addiction (Morisot and Ron, 2017), Alzheimer's disease (Yang K. et al., 2011), and pain (Yang H. B. et al., 2011; Hildebrand et al., 2016).

Fyn is composed of regulatory and catalytic domains. The regulatory domain consists of a short unique region at the N-terminus that contains myristoylation and palmitoylation sites which anchor the kinase to membranes, a proline-rich SH3 binding domain, and a phosphotyrosine-binding SH2 domain (Boggon and Eck, 2004). The catalytic domain is responsible for substrate phosphorylation and contains an autophosphorylation site (Boggon and Eck, 2004). In its inactive conformation, Fyn is phosphorylated on Tyrosine 531 (Tyr531, mouse). PhosphoTyr531[Fyn] forms an intra-molecular bond with the SH2 domain, which keeps the kinase in a closed inactive conformation (Boggon and Eck, 2004). Dephosphorylation of this site results in a conformational change, allowing the kinase to undergo autophosphorylation at Tyrosine 420 (Tyr420, mouse), leading to a fully active kinase (Boggon and Eck, 2004).

Although many of Fyn's substrates in the CNS have yet to be identified, one of the most well-characterized substrates of the kinase in the brain, is the GluN2B subunit of the N-Methyl D-Aspartate receptor (NMDAR) (Nakazawa et al., 2001; Trepanier et al., 2012). Fyn is localized in close proximity to GluN2B by interacting with the scaffolding protein RACK1 (Yaka et al., 2002; Thornton et al., 2004), and the post-synaptic density 93 (PSD93) (Nada et al., 2003; Sato et al., 2008). Once active, Fyn phosphorylates GluN2B which in turn increases the membranal retention of the subunit (Dunah et al., 2004; Prybylowski et al., 2005; Hayashi et al., 2009; Wang et al., 2010) leading to an enhancement of channel activity (Yaka et al., 2002, 2003a; Trepanier et al., 2012).

The canonical activation of Src PTKs is through growth factors-mediated activation of receptor tyrosine kinases (Bromann et al., 2004). Curiously though, Fyn is also activated in the brain through the activation of seven transmembrane Gα*s*/*olf* coupled receptors which are positively coupled to the cAMP/PKA signaling pathway. For example, the activation of the Gα*s*/*olf*-coupled Pituitary Adenylate Cyclase-Activating Peptide (PACAP) PAC1 receptors (Laburthe and Couvineau, 2002) and the dopamine D1 receptors (D1Rs) (Neve et al., 2004) activate Fyn in the hippocampus (Yaka et al., 2003a; Trepanier et al., 2012), and more recently, Mao et al. reported that Fyn is also activated by D1R activation in the rat striatum (Mao and Wang, 2015).

The striatum is the major input nucleus of basal ganglia (Kreitzer, 2009), and is divided into the ventral striatum (the nucleus accumbens, NAc) and the dorsal striatum, which is further divided into the dorsolateral and dorsomedial striatum (DLS and DMS, respectively) (Voorn et al., 2004; Nicola, 2007; Kreitzer, 2009). The three striatal regions receive different inputs, have unique peptide and receptor pattern of distribution (Kreitzer, 2009), and are part of circuitries that promote distinct behaviors including reward, locomotion, instrumental learning, habit formation, and addiction (Voorn et al., 2004; Luft and Buitrago, 2005; Yin and Knowlton, 2006; Redgrave et al., 2010; Sesack and Grace, 2010). For example, the NAc is a central core of the reward pathway (Sesack and Grace, 2010), whereas the DLS is associated with habit formation (Yin and Knowlton, 2006; Redgrave et al., 2010), and the DMS with locomotion and goal directed behaviors (Luft and Buitrago, 2005; Redgrave et al., 2010). The principal neurons of the striatum are GABAergic medium spiny neurons (MSNs) that can be divided into two populations; MSNs that selectively express D1Rs and MSNs that selectively express the dopamine D2 receptors (D2R) (Surmeier et al., 2007; Gerfen and Surmeier, 2011). D1Rs and D2Rs expressing MSNs project directly or indirectly to substantia nigra pars reticula, to promote or gate behaviors, respectively (Gerfen and Surmeier, 2011; Calabresi et al., 2014).

Dopamine is a crucial neuromodulator of striatal activity including glutamatergic signaling and synaptic plasticity (Surmeier et al., 2007; Cerovic et al., 2013). As Fyn is activated by D1R stimulation in the striatum (Mao and Wang, 2015), we **s**ought to determine whether the dopamine-dependent activation of Fyn is localized to selective substriatal regions and/or to specific neuronal populations, and if so, we aimed to identify potential mechanisms that underlie the brain region specificity of Fyn signaling.

## Materials and methods

### Materials

Rabbit anti-GAPDH, goat anti-GluN2B, rabbit anti-Fyn, and rabbit anti-a green fluorescent protein (GFP) (used for western blot analysis) antibodies were purchased from Santa Cruz Biotechnology (Santa Cruz, CA). Rabbit anti-[pY1472]GluN2B antibodies and rabbit anti-[pY418/420]Src/Fyn antibodies were purchased from Cell Signaling Technology (Beverly, MA). Rabbit anti-GFP antibodies used for immunofluorescence analysis were purchased from Abcam (Cambridge, MA). Mouse anti-Src antibodies were purchased from EMD Millipore (Temecula, CA). Mouse anti-RACK1 and mouse anti-Flotillin-1 antibodies were purchased from BD Biosciences (San Jose, CA). Mouse anti-Transferrin Receptor antibodies were purchased from ThermoFisher Scientific (Waltham, MA). Donkey anti-rabbit horseradish peroxidase (HRP), donkey anti-goat horseradish peroxidase (HRP) and donkey anti-mouse HRP-conjugated secondary antibodies were purchased from Jackson ImmunoResearch (West Grove, PA). SKF81297 and Quinpirole were purchased from R&D Systems (Minneapolis, MN). NuPAGE Bis-Tris precast gels, Alexa Fluor 488-labeled donkey anti-rabbit, Prolong Gold mounting medium, Protein G agarose and Lipofectamine 2000 Transfection Reagent were purchased from Life Technologies (Carlsbad, CA). Enhance Chemiluminescence (ECL) was purchased from GE Healthcare (Marlborough, MA). Agfa X-Ray Film was purchased from VWR (Radnor, PA). EDTA-free protease inhibitor cocktail was purchased from Roche (Indianapolis, IN). DMSO, chloroform, isopropanol, NP-40, Phosphatase inhibitor cocktails 2 and 3 were purchased from Sigma Aldrich (St. Louis, MO). Saline (0.9% sodium chloride) was purchased from Hospira, Inc. (Lake Forest, IL). Cre Recombinase was purchased from New England BioLabs (Ipswich, MA). The reverse transcription system and 2X PCR Master Mix were purchased from Promega (Madison, WI). The p24 antigen ELISA kit was purchased from Zepto Metrix (Buffalo, NY). Bicinchoninic acid (BCA)^TM^ protein assay kit was purchased from Pierce (Rockford, IL). Nitrocellulose membrane was purchased from Millipore (Billerica, MA). Cholesterol and Cholesteryl Ester Colorimetric/Fluorometric Assay Kit was purchased from BioVision (Milpitas, CA).

### Animals

Male C57BL/6J mice (6–8 weeks of age) were obtained from Jackson Laboratory (Bar Harbor, ME). The generation of Drd1-Cre-Ai14 and Drd2-Cre-Ai14 is described in Wang et al. (2015). Mouse genotypes were determined by PCR analysis of products derived from tail DNA. Animals were group housed in a 12 h/12 h light/dark cycle room, and food and water were provided *ad libitum*. All animal procedures in this report were approved by the University of California San Francisco (UCSF) Institutional Animal Care and Use Committee and were conducted in agreement with the Association for Assessment and Accreditation of Laboratory Animal Care (AAALAC, UCSF).

### Drugs preparation and *in vivo* administration

SKF81297 and Quinpirole were dissolved in 2% DMSO or saline respectively. Mice were habituated to the intraperitoneum (i.p.) administration procedure by being injected daily with saline for 3 days. On day 4, mice were systemically treated with 5 mg/kg of SKF81297, Quinpirole or vehicle (2% DMSO or saline, respectively) and the striatum was harvested 15 min later.

### Collection of brain samples

Animals were euthanized via cervical dislocation and brains were rapidly dissected on ice using a 0.5 mm brain block. The NAc, DMS, and DLS were dissected between Plates 21–26 in Franklin and Paxinos mouse atlas, third edition. Specifically, the NAc, DMS, and DLS were dissected between +1.18 and + 0.74 mm anterior to bregma. The NAc was dissected −4 to −4.75 mm below the brain surface and ±0.25 to 1.5 medial to midline. The DMS and DLS were dissected −2.25 to −3.75 mm below the brain surface and ±0.75 to 1.25 mm and ±1.75 to 2.5 mm medial to midline, respectively. Tissue was collected into 2.5 ml Eppendorf tubes and immediately homogenized in 300 μl RadioImmuno Precipitation Assay (RIPA) buffer (in mM: 50 Tris-HCl, pH 7.6, 150 NaCl, 2 EDTA, and 1% NP-40, 0.1% SDS, and 0.5% sodium deoxycholate, protease, and phosphatase inhibitor cocktails).

### Western blot analysis

Equal amounts of homogenates from individual mice (30 μg) were resolved on NuPAGE Bis-Tris gels and transferred onto nitrocellulose membranes. Blots were blocked in 5% milk-PBS, 0.1% Tween 20 for 30 min and then probed with anti-[pY1472]GluN2B (1:250), anti-[pY450/424]Fyn/Src (1:500), or anti-GAPDH (1:2,000) antibodies overnight at 4°C. Membranes were washed and probed with HRP-conjugated secondary antibodies for 2 h at room temperature. Membranes were then stripped for 30 min at room temperature in a buffer containing 25 mm Glycine-HCL and 1% (w/v) SDS, pH 3.0, and reprobed with anti-GluN2B (1:500) or anti-Fyn (1:500) antibodies. Membranes were visualized using ECL.

### Slice experiments

DMS slices (250 μm) were dissected as described above, and maintained for at least 2 h in artificial CSF (aCSF) that contained the following (in mM): 126 NaCl, 1.2 KCl, 1.2 NaH_2_PO_4_, 0.01 MgCl_2_, 2.4 CaCl_2_, 18 NaHCO_3_, and 11 glucose, and saturated with 95% O_2_/5% CO_2_ at 25°C. After recovery, slices were treated with SKF81297 (10 μM) or vehicle (DMSO 0.1%) for 15 min and homogenized in 1X immunoprecipitation (IP) buffer.

### Immunoprecipitation

Brain regions were dissected as described above, and tissues were then homogenized in IP buffer (in mM: 150 NaCl, 10 Tris-HCl pH 7.4, 1 EDTA, 1 EGTA. 1% Triton-X, protease, and phosphatase inhibitor cocktails). The homogenates were pre-cleared by incubation with protein G agarose for 1 h at 4°C. The samples were centrifuged and protein quantity was determined using the BCA^TM^ protein assay. IPs were performed by combining 1 μg of the appropriate antibody with 500 μg lysate diluted in IP buffer to a total volume of 1 ml. Following overnight incubation at 4°C, protein G agarose was added and the mixture was incubated at 4°C for 4 h. The protein G was washed extensively with IP buffer and pellets were re-suspended in 40 μl of 2x Laemmli buffer and incubated at 95°C for 10 min. The proteins in the supernatant were separated by SDS-PAGE gels and visualized by ECL.

### RT-PCR

Reverse transcription polymerase chain reaction (RT-PCR) analysis was conducted as described in Jeanblanc et al. (2009). Briefly, 400 ng of messenger RNA (mRNA) was reverse transcribed (RT) using the Reverse Transcription System Kit (Promega). The RT product was used for PCR reactions using the following primers: Fyn: (F) 5′-CATCTTCTGTCCGTGCTTCA-3′, (R) 5′-CTCAGCACTACCCCAGCTTC-3′, annealing temperature of 60°C for 30 cycles; GAPDH: (F) 5′-TGAAGGTCGGTGTGAACGGATTTGGC-3′, (R) 5′-CATGTAGGCCATGAGGTCCACCAC-3′, at annealing temperature of 62°C for 30 cycles. GFP: (F) 5′-CACATGAAGCAGCACGACTT-3, (R) 5′-CATTGTGGGCGTTGTAGTTG-3′ at annealing temperature of 60°C for 32 cycles. The PCR product was separated on 1.2% agarose gel, photographed by Image Lab, and quantified by using ImageJ.

### Immunochemistry

Mice were deeply anesthetized with Euthasol and perfused with 0.9% NaCl, followed by 4% paraformaldehyde in PBS, pH 7.4. Brains were removed, post-fixed in the same fixative for 2 h, and transferred to PBS at 4°C. On the following day, brains were transferred into 30% sucrose and stored for 3 days at 4°C. Frozen, 50 μm-thick coronal sections were cut on a cryostat (CM3050, Leica, Buffalo Grove, IL), collected in 24-well plates, and stored in PBS at 4°C. Free-floating sections containing the infusion site in the striatum were selected, permeabilized with, and blocked in, PBS containing 0.3% Triton and 5% donkey serum for 4 h. Sections were then incubated for 18 h at 4°C on an orbital shaker with anti-GFP antibodies (1:10,000) diluted in PBS, 3% BSA. Next, sections were washed in PBS then incubated for 4 h with Alexa Fluor 488-labeled donkey anti-rabbit diluted in PBS/3% BSA. After staining, sections were rinsed in PBS and cover slipped using Prolong Gold mounting medium. Images were acquired using Zeiss LSM 510 META laser confocal microscope (Zeiss MicroImaging, Jena Germany) using manufacture recommended filter configurations.

### Lipid rafts isolation

Lipid rafts were isolated as described in Gibb et al. (2011). Briefly, immediately after being collected, DMS tissues were homogenized with 20 strokes in a glass homogenizer containing 2 ml of ice-cold MES-buffered saline (MBS; in mM: 25 MES pH 6.5, and 150 NaCl containing 0.25% Triton X-100, and protease, and phosphatase inhibitor cocktails). The lysate was mixed with equal volume of 80% sucrose (w/v) in MBS, and placed in the bottom of a 14 × 89 mm clear centrifuge tube (Beckman Coulter, Brea, CA). The samples were then overlaid with 4 ml of 30% sucrose, followed by 4 ml 5% sucrose in MBS and ultracentrifuged at 200,000 g using a SW-41 Ti rotor (Beckman, Brea, CA) at 4°C for 18 h. Samples were divided into ten 1 ml fractions starting from the top of the gradient. Total protein concentration was determined using BCA ™ protein assay kit.

### Cholesterol assay

Cholesterol measurements were conducted using Cholesterol and Cholesteryl Ester Colorimetric/Fluorometric Assay Kit according to manufacturer's instruction. Briefly, striatal tissues were collected, weighed and homogenized in 200 μl cholesterol extraction solution (7 ml chloroform, 11 ml Isopropanol, and 0.1 ml NP-40). The homogenate was centrifuged at 15,000 g for 10 min. The supernatant was transferred to a new tube and liquid was evaporated by heat (50°C). The remaining fraction containing lipids was dissolved in 200 μl of cholesterol assay buffer. Ten microliters of sample was used to determine the cholesterol content. Cholesterol amounts were multiplied by 20 then divided by tissue weight to obtain μg cholesterol per mg tissue.

### Construction FLEX-shRNA Fyn

Short hairpin (sh) sequence targeting Fyn mRNA as well as the scrambled (SCR) control sequence was designed using the siRNA wizard bioinformatics program (http://www.invivogen.com/sirnawizard). shFyn sequence: 5′-GATGCTGAGAGACAGCTCC-3′; SCR sequence: 5′-GCGTCGACCAGCGATTAGA-3′.

The Cre-dependent Flip Excision (FLEX) plasmid containing two sets of loxP sites (pAAV-EF1a-DIOhChR2) was obtained from Karl Deisseroth (Stanford University, CA) (Tye and Deisseroth, 2012). The ChR2-GFP gene was substituted by the inverse CMV promoter together with the shRNA targeting *Fyn*, and the FLEX-shFyn sequence was then inserted into the pLVX-shRNA lentiviral vector. Specifically, in order to construct a lentivirus vector that expresses shRNA targeting Fyn only in the presence of Cre recombinase, first shFyn sequence (or scramble sequence) and CMV promoter (from pLL3.7 vector, sense primer: 5′-ACATTGATTATTGACTAGTTA-3′, antisense primer: 5′- GCTTATATAGACCTCCC-3′) were subcloned in a reversed orientation into pAAV-DIO-hChR2 between two sets of lox sites using NheI, AflII, and AscI sites [(shuttled in pUSEamp(+) vector (EMD Millipore (Temecula, CA)]. The fragment containing the two sets of loxP sites were excised from pAAV-DIOhChR2 plasmid and subcloned into pLVX-shRNA vector (Clontech, Mountain View, CA) by BamHI and EcoRI sites. The constructs were confirmed by sequencing (Eurofins, Alabama USA, using commercial T7 sense primer).

### Lentivirus packaging and titer determination

The pLVX-FLEX plasmid was transfected into HEK293T cells (Clontech, Mountain View, CA) along with the packaging plasmids, psPAX2 and pMD2.G, using Lipofectamine 2000. Forty-eight hours after transfection, the supernatant was collected and lentiviral particles were purified by ultra-centrifugation at 26,000 g (optimaTM LE-80K ultracentrifuge, Beckman Coulter,) for 90 min at 4°C. The titers were determined using HIV-1 p24 antigen ELISA Kit (ZeptoMetrix) per the manufacturer's instruction.

### Cell culture and *ex vivo* characterization of cre-dependent Fyn knockdown by pLVX-FLEX-shFyn

HEK293T cells were cultured in Dulbecco's Modified Eagle medium (DMEM) supplemented with 10% fetal bovine serum (FBS) and 1 × MEM non-essential amino acid solution in a 5% CO2 incubator. Cells were plated at 4 × 10^5^ cells per well on a 12-well plate. pLVX-FLEX-shFyn or pLVX-FLEX-SCR were incubated with Cre recombinase (New England Biolabs, Ipswich, MA) at the ratio of 1 μg plasmid to 4 units of Cre according to manufacturer's instruction. The plasmids were then co-transfected with pUSE-Fyn into HEK293T cells at the ratio of 1.5 μg pLVX-FLEX to 0.5 μg pUSE-Fyn per well using Lipofectamine. Cells were imaged for GFP expression and harvested 48 or 72 h after transfection for PCR or western blot analyses, respectively.

### Lentiviral infection of the DMS

Mice were anesthetized using a mixture of ketamine (120 mg/kg) and xylazine (8 mg/kg). Bilateral microinfusions were made using stainless steel injectors (33 gauge, Small Parts) into the DMS (the stereotaxic coordinates were anterioposterior +1.55 mm and +1.2 mm from bregma; mediolateral ±1.25 mm from bregma and dorsoventral −3.2 and −3.5 mm from the skull surface). Animals were infused with the lentivirus (1.2 μl/injection site with four sites of injection per hemisphere) at a concentration of 2 × 10^7^ pg/ml at an injection rate of 0.1 μl/min (Lasek et al., 2010). After each infusion, the injectors were left in place for an additional 12 min to allow the virus to diffuse.

### Statistical analysis

All data are expressed as mean ± SEM. The optical density of the relevant band was quantified using NIH ImageJ 1.44c software (NIH). Data were analyzed using the appropriate statistical test, including two-tailed unpaired *t*-test, two-way analysis of variance (ANOVA) as detailed in the figure legends. Significant main effects and interactions of the ANOVAs were further investigated with the Tukey's multiple comparison *post-hoc* test. Data are expressed as mean ± S.E.M and statistical significance was set at *p* < 0.05.

## Results

### Dopamine D1 receptor-mediated activation of Fyn and GluN2B phosphorylation is restricted to the DMS

**Figure 1:**
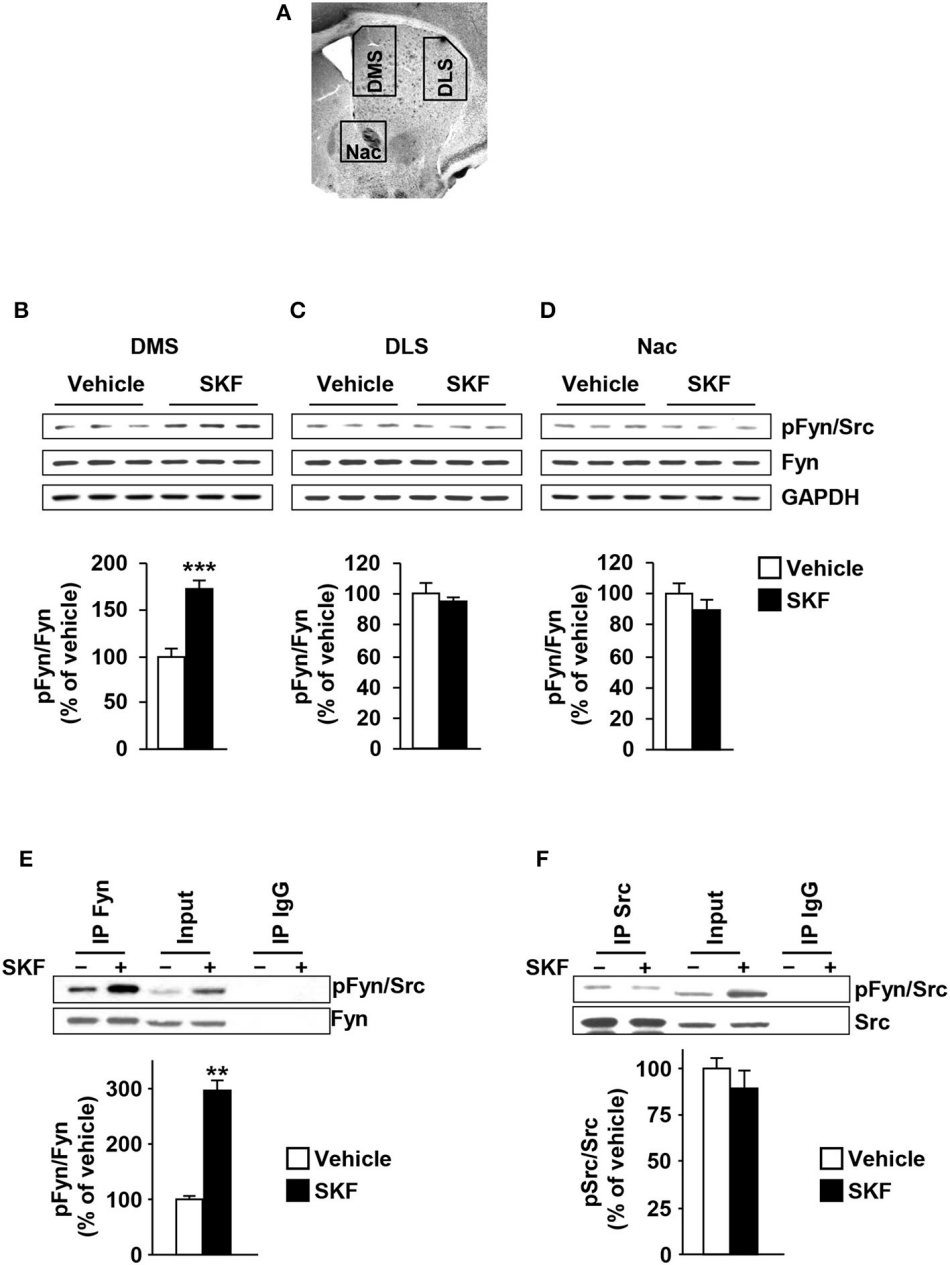
Dopamine D1R stimulation activates Fyn but not Src in the DMS but not in the DLS or NAc. **(A)** Schematic representation of DMS, DLS, and NAc dissection. **(B–D)** SKF81297 (SKF, 5 mg/kg) or vehicle (2% DMSO) was systemically administered (i.p.). The DMS **(B)**, DLS **(C)**, and NAc **(D)** were collected 15 min after drug administration. The level of Tyr420 phosphorylation (pFyn) (upper panel), Fyn (middle panel), and GAPDH, which was used as a loading control (bottom panel) were determined by western blot analysis. Data are presented as the densitometry value of the pFyn divided by the densitometry values of Fyn ± SEM and expressed as % of vehicle. **(E,F)** Mouse striatal sections were treated with SKF81297 (SKF, 10 μM) or vehicle (0.1% DMSO) for 15 min. The DMS was dissected and Fyn or Src were immunoprecipitated using anti-Fyn (IP Fyn) **(E)** or anti-Src (IP Src) **(F)** antibodies. Mouse IgG was used as a negative control (IP IgG). Fyn and Src immunoreactivity (lower panels) as well as Tyr417/420[Src/Fyn] phosphorylation (upper panels) were determined by westernblot analysis. Protein levels in the total lysates were measured in parallel (Input). Two-tailed *t*-test. **(B–D)** ^***^p < 0.001, *n* = 6 mice per treatment. **(E,F)** ^**^p < 0.01, *n* = 3 mice per treatment.

Fyn was reported to be activated in the rat striatum in response to systemic administration of the D1R agonist, SKF81297 (Mao and Wang, 2015). Thus, we determined whether the D1R-dependent activation of Fyn is localized in one or more substriatal regions. To do so, mice were systemically administered with SKF81297 (5 mg/kg) or vehicle (2% DMSO in saline), 15 min later animals were sacrificed, the DMS, DLS and NAc were dissected (Figure 1A), and Fyn autophosphorylation (and thus activation) in the three brain regions was determined. Surprisingly, we found that SKF81297 administration activates Fyn only in the DMS [Figure 1B, *t*_(10)_ = 6.285, *p* < 0.001] but not in the DLS or NAc [Figures 1C,D, DLS (*t*_(10)_ = 0.7544, *p* = 0.46); NAc (*t*_(10)_ = 1.119, *p* = 0.28)].

The antibodies that recognize the autophosphorylation site of Fyn also recognize the autophosphorylation site of Src, a member of the none-receptor PTKs which is also highly expressed in the CNS (Salter and Kalia, 2004). Therefore, we determined whether SKF81297 administration specifically activates Fyn in the DMS. To do so, mouse DMS were dissected from striatal sections and treated with SKF81297 (10 μM) or vehicle *ex vivo* for 15 min, Fyn and Src were immunoprecipitated (IPed) and kinase activation was tested using the anti-phosphoTyr418/420[Src/Fyn] antibodies. As shown in Figures 1E,F, Fyn but not Src is activated in the DMS in response to D1R stimulation.

**Figure 2:**
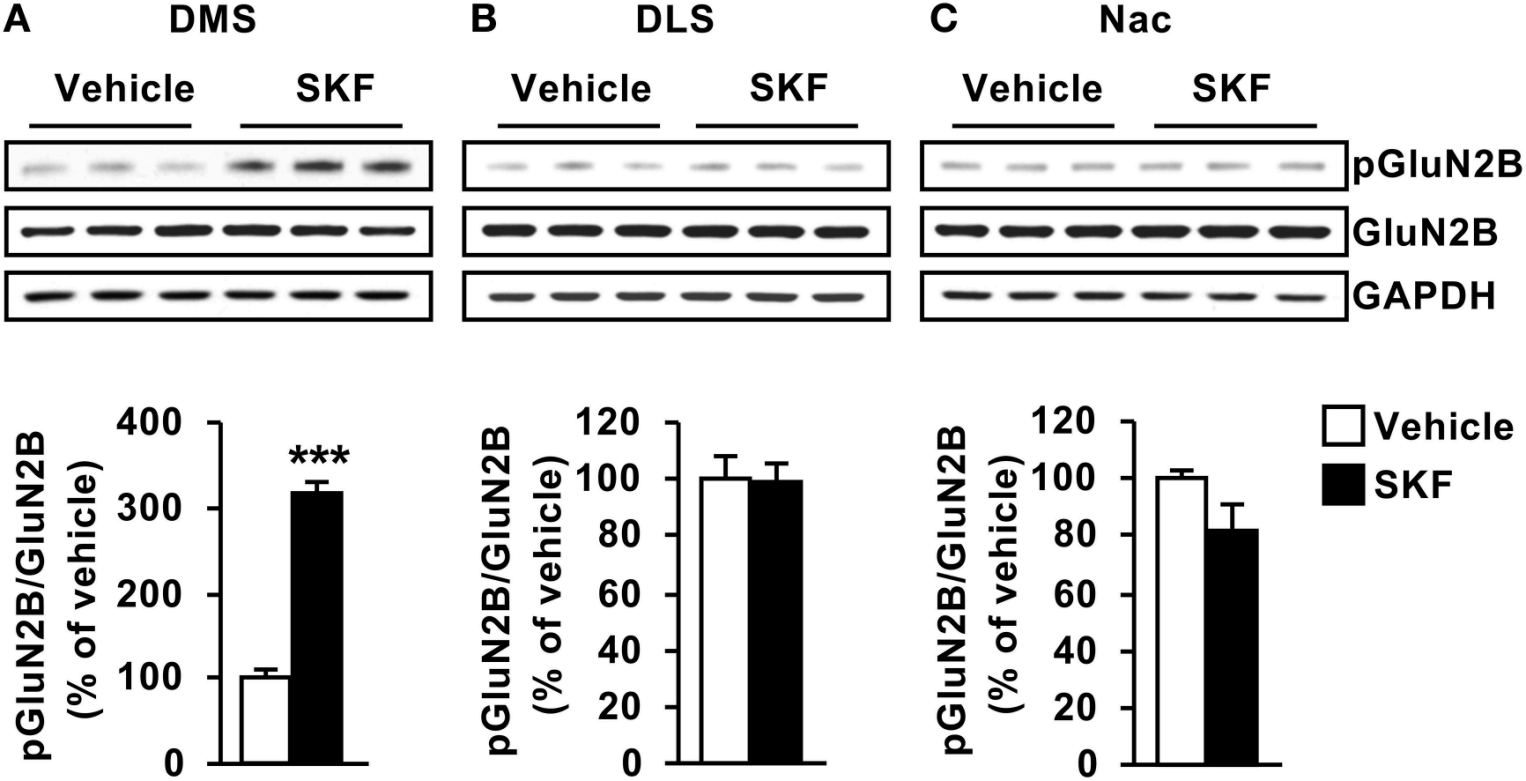
D1R-dependent GluN2B phosphorylation is detected in the DMS but not in the DLS or NAC. SKF81297 (SKF, 5 mg/kg) or vehicle (2% DMSO) was systemically administered (i.p.). The DMS **(A)**, DLS **(B)**, and NAc **(C)** were collected 15 min after drug administration. The level of Tyr1472[GluN2B] phosphorylation (pGluN2B, top panels), GluN2B (middle panels), and GAPDH (lower panels) were determined by western blot analysis. Data are presented as the mean densitometry value of the pGluN2B divided by the mean densitometry values of the total GluN2B ± SEM and expressed as % of vehicle. Two-tailed *t*-test. ^***^p < 0.001, *n* = 5–6 mice per treatment.

As GluN2B is a substrate of Fyn (Trepanier et al., 2012), we next examined whether the profile of D1R-mediated activation of Fyn in the striatum corresponds to the profile of GluN2B phosphorylation. To do so, the same samples used to detect Fyn phosphorylation were used to measure the levels of GluN2B phosphorylation, and as shown in Figure 2, similar to the pattern of Fyn activation, GluN2B was phosphorylated in response to D1R activation only in the DMS but not in the DLS or NAc [Figure 2A, DMS (*t*_(10)_ = 11.44, *p* < 0.001); Figure 2B, DLS (*t*_(10)_ = 0.0521, *p* = 0.95); Figure 2C NAc (*t*_(9)_ = 1.603, *p* = 0.14)]. Together, these findings suggest that D1R stimulation leads to Fyn activation specifically in the DMS, which results in a concomitant GluN2B phosphorylation.

**Figure 3:**
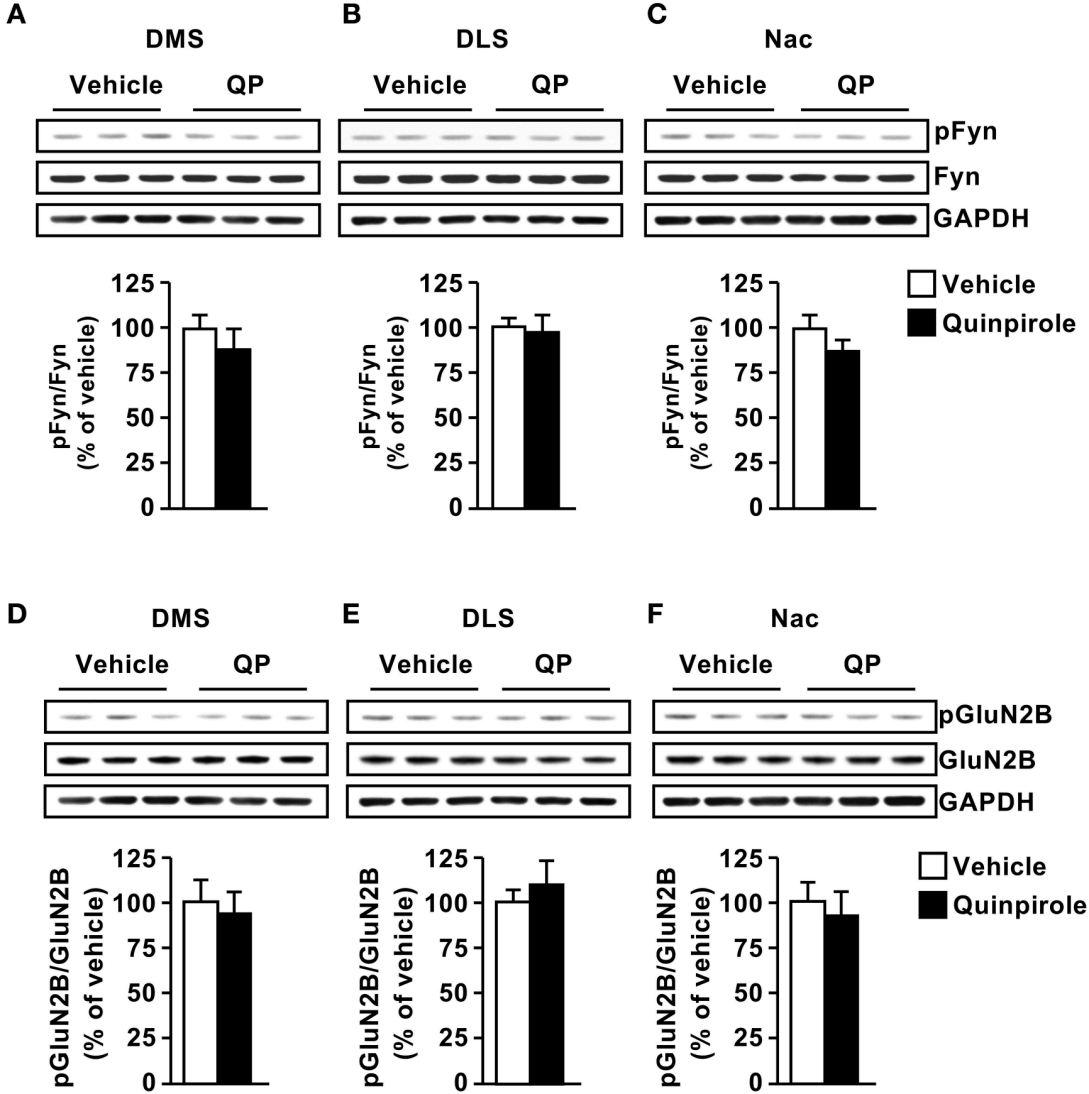
Dopamine D2 receptor stimulation does not alter Fyn activation or GluN2B phosphorylation. Quinpirole (QP; 5 mg/kg) or saline was systemically administered (i.p.). The DMS **(A,D)**, DLS **(B,E)**, and NAc **(C,F)** were collected 15 min after drug administration. The level of Tyr420[Fyn] phosphorylation (pFyn) (upper panels), Fyn (middle panels) (**A–C**), Tyr1472[GluN2B] phosphorylation (pGluN2B, top panels), GluN2B (middle panels) (**D–F**) were measured by western blot analysis. The immunoreactivity of GAPDH (bottom panels) was used as a loading control (bottom panel). Data are presented as the mean densitometry value of the phosphorylated protein divided by the mean densitometry values of the total protein ± SEM and expressed as % of vehicle. Two-tailed *t*-test. *n* = 5–6 mice per treatment group.

Next, we tested whether D2R stimulation activates the Fyn/GluN2B signaling in the 3 striatal subregions. To do so, mice were systemically administered with the D2R agonist Quinpirole (5 mg/kg) or vehicle (saline), 15 min later the striatal regions were dissected and Fyn and GluN2B phosphorylation were measured. We did not detect changes in phosphorylation of the kinase or its substrate in the three striatal subregions [Figure 3A, DMS (*t*_(10)_ = 0.8901, *p* = 0.39); Figure 3B, DLS (*t*_(10)_ = 0.2982, *p* = 0.77); Figure 3C NAc (*t*_(10)_ = 1.299, *p* = 0.22); Figure 3D, DMS (*t*_(10)_ = 0.4541, *p* = 0.65); Figure 3E, DLS (*t*_(10)_ = 0.6255, *p* = 0.54); Figure 3F NAc (*t*_(9)_ = 0.4881, *p* = 0.63)].

### Generation of a lentivirus expressing shFyn in a cre-dependent manner

**Figure 4:**
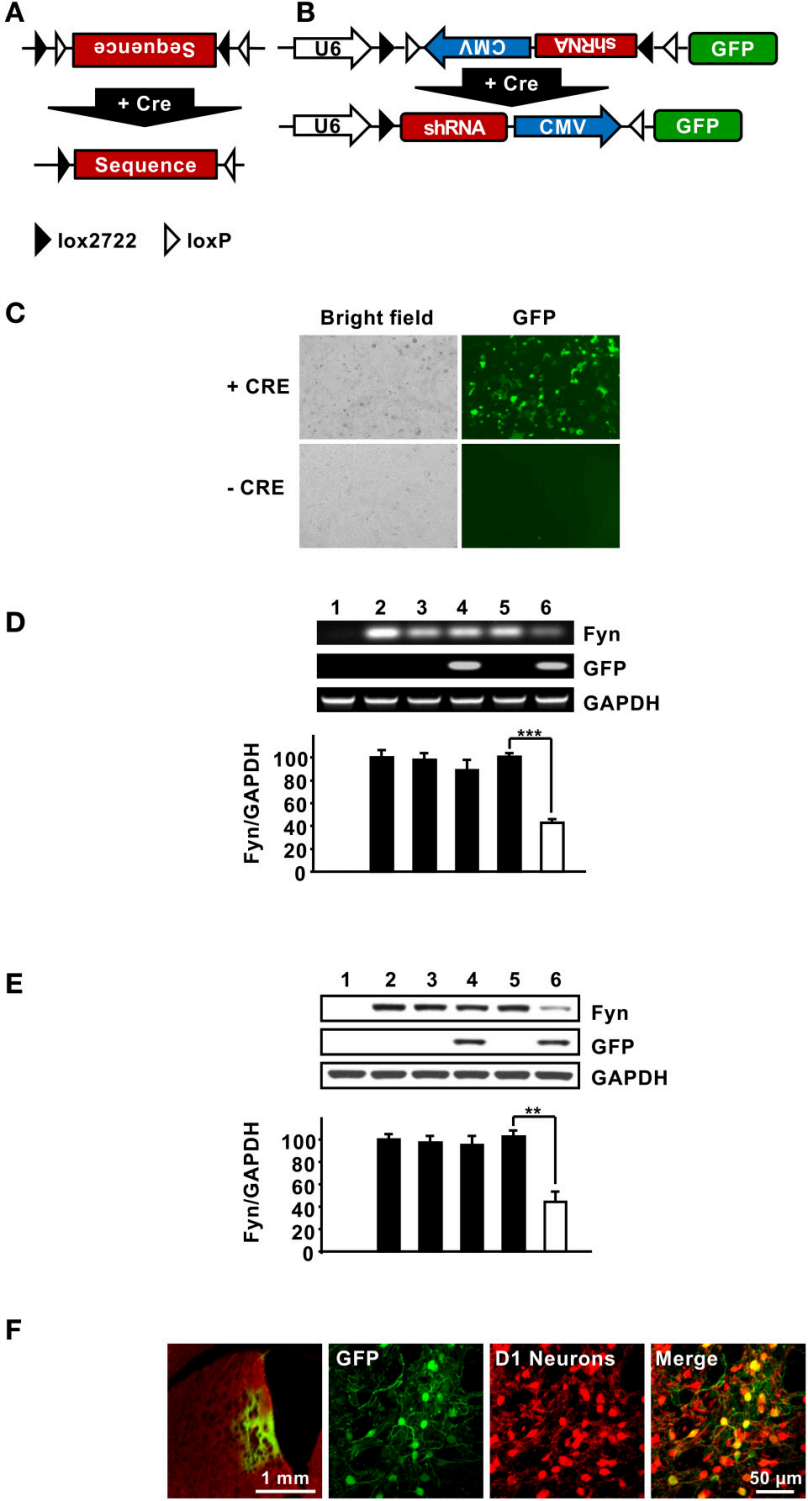
Design and characterization FLEX-Cre system. **(A,B)** Schematic representation of the FLEX vector which encodes a double-Floxed, inverted shRNA and GFP. In the presence of Cre, a FLip-EXcision (FLEX) switch occurs, leading to a U6 promoter-driven expression of the gene-specific shRNA, and a CMV promoter- driven expression of GFP. **(C)** The FLEX-shFyn plasmid was incubated with (+ Cre) or without (− Cre) Cre recombinase *in vitro* and infection was evaluated by staining cells with anti-GFP antibodies (right panels). **(D,E)** Characterization of Cre-driven Fyn knockdown in HEK293T cells. **(D,E)** FLEX-shFyn or FLEX-SCR plasmids were pretreated with (+ Cre) or without Cre recombinase then co-transfected with Fyn plasmid as indicated. mRNA expression **(D)** and protein levels **(E)** were analyzed by RT-PCR and western blot analysis, respectively. 1. pUSE-empty plasmid, 2. pUSE-Fyn, 3. pUSE-Fyn + pLVX-FLEX-SCR, 4. pUSE-Fyn + pLVX-FLEX-SCR + Cre, 5. pUSE-Fyn + pLVX-FLEX-shFyn, 6. pUSE-Fyn + pLVX-FLEX-shFyn + Cre. Two-tailed *t*-test. ^***^p < 0.001; ^**^p < 0.01. **(D)** *n* = 3, **(E)** *n* = 4. **(F)** Verification of Cre-dependent pLVX-FLEX-shFyn infection *in vivo*. The DMS of Drd1-Cre-Ai14 mice was infected with Ltv-FLEX-shFyn (2 × 10^7^ pg/ml). Colocalization of infected neurons (GFP, green, left panels) with TdTomato (D1R neurons, red, right panels) was determined 3 weeks later. Merged image, right panel. Left panel, 5X; middle and right panels, 20X.

The data described above suggest that Fyn is a transducer of the dopamine D1R system in the DMS. Striatal neurons can be segregated into two major classes; MSNs that express D1Rs and MSNs that express D2Rs (Gerfen and Surmeier, 2011). We therefore hypothesized that the pathway is activated only in DMS D1R-receptor expressing MSNs. To differentiate between Fyn expressed in D1R vs. D2R MSNs, we developed a method in which short hairpin RNA (shRNA) that targets a specific gene is expressed only in the presence of Cre-recombinase (Cre). Specifically, we adapted a method described by Atasoy et al. (2008), and created a lentiviral vector in which the inverted sequences of the shRNA targeting Fyn and the CMV promoter are adjacent two sets of loxP sites (Figure 4A). The viral vector also encodes GFP as an indicator of Cre-dependent infection. In the presence of Cre, a FLip-EXcision (FLEX) switch recombination occurs in two orthogonal recombination sites: (1) inversion, followed by (2) excision leading to a U6 promoter-driven expression of the specific shRNA, and a CMV promoter-driven expression of GFP (Figure 4B). In order to assess the efficacy of Cre-specific knockdown of Fyn, the FLEX-shFyn plasmid as well as a plasmid expressing a scramble sequence control (Flex-SCR), were pretreated *in vitro* with Cre recombinase as described in Cantor and Chong (2001). HEK293T cells were then transfected with the inverted (i.e., Cre-pretreated) or uninverted (Cre-untreated) plasmid along with a plasmid that expresses Fyn and transfection was monitored using immunohistochemistry. As shown in Figure 4C, GFP was detected only in the presence of Cre, and accordingly the mRNA (Figure 4D) and protein levels of Fyn (Figure 4E) were reduced only in cells transfected with Cre+FLEX-shFyn [Figure 4D, (*t*_(4)_ = 12.13, *p* < 0.001); Figure 4E, (*t*_(6)_ = 5.514, *p* < 0.01)].

Next, FLEX-shFyn was packaged into a lentivirus (Ltv-FLEX-shFyn) and the efficacy of *in vivo* infection in D1R DMS neurons was evaluated by using Drd1-Cre-Ai14 mice which express Cre recombinase and the red fluorescence protein, tdTomato specifically in D1R neurons (Shuen et al., 2008). Viral infection was determined by immunostaining of GFP, and as shown in Figure 4F, we found that D1R positive neurons (red) also expressed GFP (green). The absence of GFP in cells without Cre indicates that the virus only infected D1R expressing neurons. Together these data show that -shFyn is expressed in the DMS neurons of mice only when Cre is present.

### The Fyn/GluN2B pathway is activated in D1R-expressing neurons in the DMS

**Figure 5:**
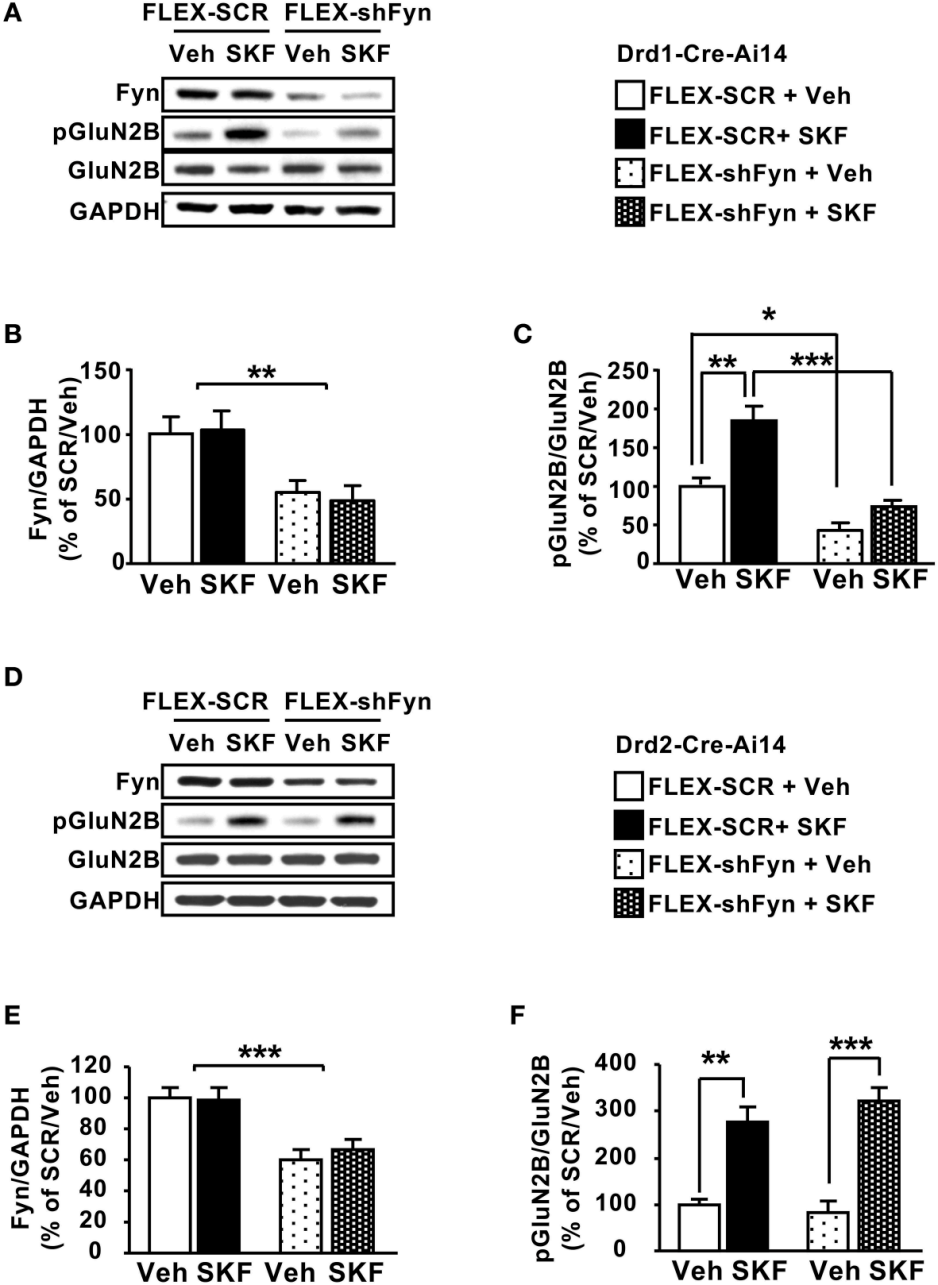
Knockdown of Fyn in DMS D1R but not in D2R neurons attenuates SKF81297-dependent GluN2B phosphorylation. **(A–C)** The DMS of Drd1-Cre-Ai14 mice **(A–C)** or Drd2-Cre-Ai14 **(D–F)** was infected with Ltv-FLEX-shFyn or Ltv-FLEX-SCR (2 × 10^7^ pg/ml). Four weeks later and 15 min before the dissection, animals received a systemic administration of SKF81297 (SKF, 5 mg/kg) or vehicle (Veh, 2% DMSO). The levels of Fyn (**A,B,D,E**), phosphoTyr1472[GluN2B] (pGluN2B) and GluN2B (**A,C,D,F**) as well as GAPDH were analyzed by western blot analysis. **(A,D)** Representative images of the western blot analysis. **(B,C)** Quantification of Fyn knockdown **(B)** and GluN2B phosphorylation **(C)** in Drd1-Cre-Ai14 mice. Data are presented as the mean densitometry value of Fyn divided by the mean densitometry values of GAPDH ± SEM **(B)** or as the mean densitometry value of pGluN2B divided by the mean densitometry values of the total GluN2B ± SEM **(C)** and expressed as % of the mean value of FLEX-SCR/Vehicle. **(B)** Two-way ANOVA showed a main effect of shFyn [*F*_(1, 12)_ = 9.507, *p* = 0.0095], no effect of SKF treatment [*F*_(1, 12)_ = 0.03439, *p* = 0.85], and no interaction [*F*_(1, 12)_ = 0.1446, *p* = 0.71]. **(C)** Two-way ANOVA showed a main effect of shFyn [*F*_(1, 12)_ = 49.24, *p* < 0.001], a main effect of SKF treatment [*F*_(1, 12)_ = 26.24, *p* = 0.0003], but no interaction [*F*_(1, 15)_ = 6.36, *p* = 0.023], and *post-hoc* Tukey's multiple comparison test showed a significant effect of SKF within the FLEX-SCR groups (SCR/Veh vs. SCR/SKF, *p* < 0.01), and a significant effect of shFyn within the vehicle groups (SCR/Veh vs. shFyn/Veh, *p* < 0.05) and within the SKF-treated groups (SCR/SKF vs. shFyn/SKF, *p* < 0.001)**. (E,F)** Quantification of Fyn knockdown **(E)** and GluN2B phosphorylation **(F)** in Drd2-Cre-Ai14 mice. Data are presented as the mean densitometry value of Fyn divided by the mean densitometry values of GAPDH ± SEM **(E)**, or as the mean densitometry value of pGluN2B divided by the mean densitometry values of the total GluN2B ± SEM **(F)** and expressed as % of the mean value of Lenti-FLEX-SCR/Vehicle. **(E)** Two-way ANOVA showed a main effect of shFyn [*F*_(1, 16)_ = 26.06, *p* = 0.0001], no effect of SKF treatment [*F*_(1, 16)_ = 0.1649, *p* = 0.69], and no interaction [*F*_(1, 16)_ = 0.3032, *p* = 0.58]. **(F)** Two-way ANOVA showed a main effect of SKF treatment [*F*_(1, 16)_ = 63.21, *p* < 0.001] but no main effect of shFyn [*F*_(1, 16)_ = 0.297, *p* = 0.593], and no interaction [*F*_(1, 16)_ = 1.506, *p* = 0.238], and *post-hoc* Tukey's multiple comparison test showed a significant effect of SKF within the FLEX-SCR groups (SCR/Veh vs. SCR/SKF, *p* < 0.01) and within the FLEX-shFyn groups (shFyn/Veh vs. shFyn/SKF81297, *p* < 0.001). ^*^p < 0.05, ^**^p < 0.01, and ^***^p < 0.001. **(A–C)** *n* = 4 and **(D–F)** *n* = 5.

Next, we tested whether knockdown of Fyn in DMS D1R neurons alters SKF81297-dependent GluN2B phosphorylation. To do so, the DMS of Drd1-Cre-Ai14 mice was infected with Ltv-FLEX-shFyn or a Ltv-FLEX-SCR control. Four weeks later animals were administered systemically with SKF81297 (5 mg/kg) or vehicle, and the DMS was harvested 15 min later. As shown in Figures 5A,B, the protein levels of Fyn were significantly reduced in Drd1-cre-Ai14 mice infected with Ltv-FLEX-shFyn compared to animals infected with Ltv-FLEX-SCR [*F*_(1, 12)_ = 9.507, *p* < 0.01]. Importantly, as shown in Figures 5A,C, SKF81297 administration increased GluN2B phosphorylation in the DMS of Drd1-Cre-Ai14 mice infected with Ltv-FLEX-SCR (FLEX-SCR/Veh vs. FLEX-SCR/SKF, *p* < 0.01), which was abolished in mice infected with Ltv-FLEX-shFyn (FLEX-SCR/SKF vs. FLEX-shFyn/SKF, *p* < 0.001). Next, we tested whether Fyn-dependent phosphorylation of GluN2B is specifically localized in DMS D1R expressing neurons. To do so, the DMS of Drd2-Cre-Ai14 mice were infected with Ltv-FLEX-shFyn or Ltv-FLEX-SCR, 4 weeks later, animals were administered systemically with SKF81297 or vehicle and the DMS was removed 15 min later. We found that similar to what we obtained following the infection of the virus in the DMS of Drd1-Cre-Ai14 mice, Fyn protein level was reduced in Drd2-Cre-Ai14 mice infected with Ltv-FLEX-shFyn compared to animals infected with Ltv-FLEX-SCR [Figures 5D–F; *F*_(1, 16)_ = 26.06, *p* < 0.001]. Administration of SKF81297 produced a similar increase in GluN2B phosphorylation in the DMS of Drd2-Cre-Ai14 mice infected with either Ltv-FLEX-SCR or Ltv-FLEX-shFyn [Figures 5D,E; FLEX-SCR/Veh vs. FLEX-SCR+SKF, *p* < 0.01; FLEX-shFyn/Veh vs. FLEX-shFyn/SKF, *p* < 0.001]. Together, these results indicate that activation of the Fyn/GluN2B pathway by SKF81297 is specifically localized to D1R DMS neurons.

### RACK1 scaffolds GluN2B and Fyn in the DMS but not in the DLS or NAc

**Figure 6:**
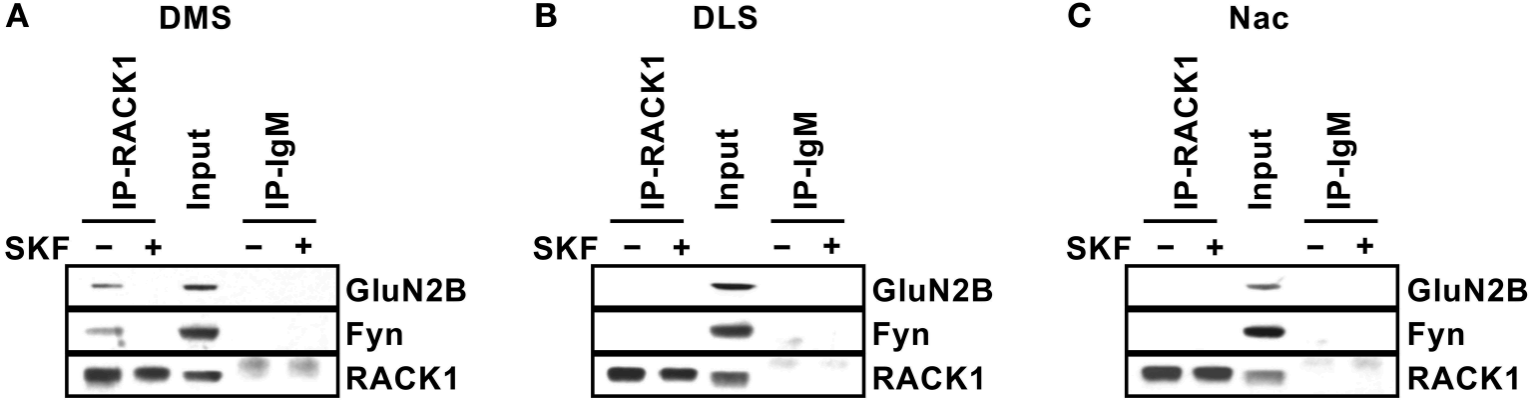
Differential compartmentalization of Fyn and GluN2B in the DMS, DLS, and NAc. Striatal slices were treated with SKF81297 (SKF, 10 μM) or vehicle (0.1% DMSO) for 15 min, followed by dissection of the DMS **(A)**, DLS **(B)**, and NAc **(C)**. The tissue was then lysed and RACK1 was IPed using anti-RACK1 antibodies (IP RACK1) and co-IP of Fyn and GluN2B was evaluated. Mouse IgM was used as a negative control (IP IgM) and protein levels in the total lysates were measured in parallel (Input). Immunoreactivity of RACK1 (lower panels), Fyn (middle panels) and GluN2B (upper panels) was detected by western blot analysis. *n* = 3.

We then explored the potential mechanism(s) for the specificity of Fyn signaling in the DMS. We previously showed that in the hippocampus, Fyn is compartmentalized to GluN2B via the scaffolding protein RACK1, which forms a bridge between the two proteins (Yaka et al., 2002). We further showed that *ex vivo* activation of the cAMP/PKA pathway in hippocampal preparations either by application of the adenylate cyclase activator, forskolin, or via the activation of the PACAP PAC1R results in the dissociation of the complex enabling Fyn to phosphorylate GluN2B (Yaka et al., 2003a). We therefore postulated that Fyn is compartmentalized to GluN2B in the DMS but not in the DLS and NAc. To test this possibility, striatal sections were dissected and treated with SKF81297 (10 μM) or vehicle for 15 min. RACK1 was then IPed, and the co-IP of Fyn and GluN2B were measured. As shown in Figure 6, under basal condition, the Fyn/RACK1/GluN2B complex was detected only in the DMS but not in the DLS or NAc, and similar to what we previously observed in the hippocampus (Yaka et al., 2003b), activation of the cAMP/PKA pathway in the DMS led to the dissociation of the complex (Figure 6). Together, these data suggest that the association of Fyn and GluN2B with RACK1 is a prerequisite for kinase activation.

### Differential distribution of Fyn and RACK1 in lipid rafts in the dorsal striatum

**Figure 7:**
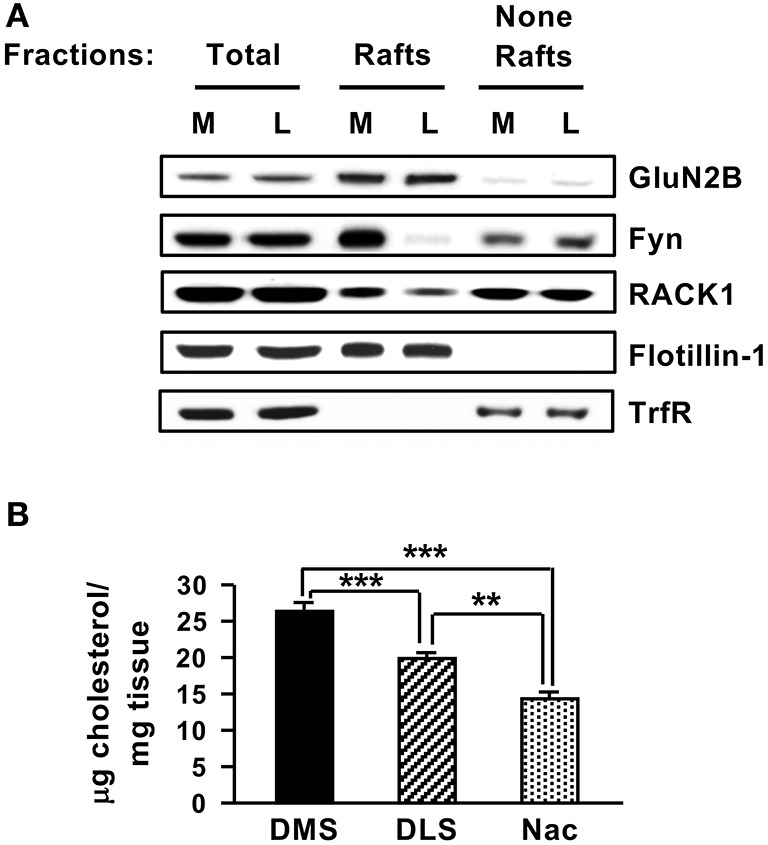
Striatal subregions differ in lipid rafts composition and cholesterol content**. (A)** The DMS (M) and DLS (L) were dissected and subjected to lipid rafts fractionation using sucrose gradient. The upper, light weight, fractions (3–4) containing lipid rafts and the bottom, heavy weight fractions (none-rafts) (8–9) were pulled together. Samples were separated on SDS-PAGE gel, and the presence of GluN2B, Fyn, and RACK1 in or out of lipid rafts was determined by western blot analysis. The rafts marker Flotillin-1 and the non-rafts marker Transferrin receptor (TrfR) were used to validate the purity of the lipid rafts fractionation. **(B)** The DMS, DLS, and NAc were dissected and were subjected to cholesterol content analysis. Data are presented as the mean cholesterol value divided by tissue weight. Two-tailed *t*-test. ^***^p < 0.001 DMS vs. DLS, ^***^p < 0.001 DMS vs. NAc, ^**^p < 0.01 DLS vs. NAc, **(A)** *n* = 3, (**B**) *n* = 6 or 7.

As shown above, in the DMS but not in the other striatal regions, Fyn and GluN2B are compartmentalized in close proximity with each other through their interaction with the scaffolding RACK1. We were therefore interested to identify a potential mechanism for the substriatal specificity of complex formation. Lipid rafts are membranal microdomains which are enriched in cholesterol and glycosphingolipids, that serve as signaling platforms by including or excluding certain receptors and signaling proteins such as Src PTKs (Simons and Toomre, 2000; Allen et al., 2007). Both GluN2B (Besshoh et al., 2005; Delint-Ramirez et al., 2010) and Fyn (Kramer et al., 1999; Filipp et al., 2003; Maksumova et al., 2005; Pereira and Chao, 2007; Vacaresse et al., 2008; Gibb et al., 2011) were shown to localize to rafts in various cell types, and we previously reported that Fyn is localized to lipid rafts in the DMS (Gibb et al., 2011). We therefore tested whether the substriatal specificity of complex formation could be due to differences in the lipid rafts composition. To do so, we compared the localization of Fyn, RACK1 and GluN2B in lipid rafts in the DMS vs. the DLS. We found that although the total amounts of Fyn, RACK1, and GluN2B were similar in the DMS and DLS, marked differences were detected in the localization of Fyn and RACK1 in the lipid raft fraction (Figure 7A). Specifically, a high level of Fyn was detected in the lipid rafts of the DMS whereas only negligible amounts of Fyn were detected in the DLS (Figure 7A). RACK1 levels in the lipid rafts fraction in the DMS were also higher as compared to the DLS, however, the differences were less robust (Figure 7A).

Finally, as cholesterol is a major components of lipid rafts (Simons and Toomre, 2000), we tested whether the DMS, DLS, and NAc differ in cholesterol content. Interestingly, as shown in Figure 7B, significant differences were observed in the cholesterol levels in the three striatal regions with higher cholesterol levels detected in the DMS vs. the DLS and NAc [Figure 7B, DMS vs. DLS (*t*_(12)_ = 4.412, *p* < 0.001); DMS vs. NAC (*t*_(11)_ = 7.687, *p* < 0.001); DLS vs. NAC (*t*_(11)_ = 4.373, *p* < 0.01)]. Together, these data raise the possibility that differences in cholesterol levels in the substriatal regions may shape the lipid rafts composition of Fyn and RACK1 which in turn may contribute, at least in part, to the differences in the Fyn/RACK1/GluN2B complex formation which is a prerequisite for Fyn activation.

## Discussion

Here, we present data to suggest that the D1R/Fyn/GluN2B signaling pathway is localized to a specific subregion of the striatum, the DMS, and that within the DMS the signaling cascade is localized to D1R-expressing MSNs. We further provide data indicating that the substriatal region specificity of the D1R/Fyn/GluN2B pathway is determined by the compartmentalization of Fyn and GluN2B with the scaffolding protein RACK1, and by the localization of Fyn in lipid rafts.

We provide data to suggest that D1R but not D2R stimulation activates Fyn in the DMS. Since striatal interneuron neurons express D5Rs (Rivera et al., 2002), we cannot exclude the possibility that the Fyn signaling pathway is also activated via dopamine interacting with D5R. In addition, as we only used one time point of SKF81297 treatment, a detailed time course is required, and we cannot exclude the possibility that D2R stimulation alters Fyn activity at a later time point.

D1R stimulation activates the cAMP/PKA pathway (Neve et al., 2004), and although the link between D1R/PKA signaling and Fyn has been well-established (Trepanier et al., 2012), the specific mechanism by which Fyn is activated by PKA remains unclear. Lombroso and colleagues showed that the activity of STriatal Enriched Protein Tyrosine Phosphatase (STEP), the phosphatase responsible for Fyn inactivation in the CNS (Goebel-Goody et al., 2012), is inhibited by PKA phosphorylation (Goebel-Goody et al., 2012). PKA-dependent inhibition of STEP accounts for the maintenance of Fyn activity, however, it does not account for the initiation of Fyn activation, i.e., the dephosphorylation of the inhibitory site Tyr530, and the autophosphorylation of the Tyr420 site. We show herein that Fyn and GluN2B associate with the scaffolding protein RACK1 in the DMS of vehicle treated mice, and that D1R stimulation by SKF81297 causes the dissociation of the molecular complex. We previously showed that RACK1 acts as an “inhibitory” scaffolding protein which localizes Fyn to GluN2B but prevents the activation of the kinase under basal conditions (Yaka et al., 2002). Our data suggest that similar to what was previously observed in the hippocampus (Yaka et al., 2003a), Fyn activation by PKA is mediated in part through the dissociation of the kinase from RACK1.

Fyn activity was unaltered in the three striatal regions in response to the activation of the D2R. Surprisingly, recent studies by Mao and colleagues suggest that systemic administration of the D2R inhibitor, Eticlopride, increased the levels of Fyn activity the striatum (Mao and Wang, 2016), as well as in the in the medial prefrontal cortex (mPFC) (Mao and Wang, 2017). These data suggest that the activation of D2Rs reduces the basal levels of active Fyn. The difference between our data and Mao and colleagues could be due to differences in the basal level of Fyn activity which were low in our studies and higher in (Mao and Wang, 2016), Mao and Wang (2017). It should be noted that high basal level of phosphorylation could be due to oxidative stress and/or ischemia which may not be relevant to the physiological condition.

The activation of Fyn signaling through the stimulation of D1R has been well-established (Yaka et al., 2003a; Trepanier et al., 2012; Mao and Wang, 2015), however, our study is the first to prove that this pathway is indeed localized to D1R expressing neurons. We did so, by developing a FLEX system that enables the downregulation of genes within subpopulations of neurons. Our study therefore puts forward the use of FLEX for the study of signaling molecules in subpopulations of cells. This system is especially useful in cases in which Floxed mice are not available, as in the case of Fyn. Finally, the FLEX system can be easily adapted for Cre-dependent overexpression of genes.

It is intriguing that the Fyn/GluN2B signaling pathway is localized to the DMS even though all three striatal subregions are composed primarily of similar type of neurons (Surmeier et al., 2007; Gerfen and Surmeier, 2011), and even though the protein levels of Fyn and GluN2B in the three striatal regions are similar. Interestingly, Fyn is compartmentalized to GluN2B via RACK1 only in the DMS but not in the DLS or NAc. Similarly, RACK1 scaffolds Fyn to GluN2B in the hippocampus but not in the cortex even though the majority of neurons in both brain regions are the same i.e. pyramidal neurons (Yaka et al., 2003b). We previously showed that RACK1 links GαsPCRs with GluN2B and Fyn in the hippocampus by forming homo and hetero-dimers (Thornton et al., 2004). The D1R and the NMDARs were shown to form a complex (Cepeda and Levine, 2006). Thus, it would be of interest to test whether RACK1 specifically bridges between the GluN2B-containing NMDARs and D1Rs in the DMS.

Lipid rafts are highly specialized microdomains at plasma membranes enriched in sphingolipids and cholesterol, glycophosphatidylinositol (GPI) linked receptors as well as numerous signaling proteins, adaptor proteins (Simons and Toomre, 2000). Lipid rafts enable the tight orchestration of signal transduction cascades in various cell types including in the CNS (Simons and Toomre, 2000; Allen et al., 2007). The localization of Fyn in lipid rafts is well-established (Kramer et al., 1999; Maksumova et al., 2005; Pereira and Chao, 2007; Vacaresse et al., 2008; Gibb et al., 2011). Surprisingly, we found that Fyn is localized within rafts in the DMS, and outside of rafts in the DLS. Previous studies have noted that the consequences of the activation of enzymes in vs. outside of rafts, are distinct. For example, the small G protein, H-Ras is localized within lipid rafts, however, once GTP bound, H-Ras moves out of the rafts in order to activate is downstream effector, Raf (Prior et al., 2001). The BDNF receptor, TrkB, moves into lipid rafts upon binding to BDNF, in a mechanism that requires Fyn (Pereira and Chao, 2007). Therefore, it is plausible that Fyn in the DMS phosphorylates GuN2B in rafts, whereas Fyn in the DLS for example, phosphorylates substrates outside of rafts resulting in different biological consequences.

Cholesterol is an essential component of cell membranes in general and of lipid rafts in particular (Simons and Toomre, 2000; Ikonen, 2008). We found that the cholesterol content in the three striatal regions is different with the DMS having the highest levels of cholesterol and the NAc the lowest. The DMS, DLS, and NAc differ in receptor distribution, synaptic plasticity, inputs, and outputs projections and behavioral responses (Partridge et al., 2000; Gerdeman et al., 2002; Voorn et al., 2004; Yager et al., 2015), and here we add another parameter that differentiates the three substriatal regions, i.e., the cholesterol content. Although further experiments are needed, it is plausible that high level of cholesterol in the DMS contributes to Fyn localization in rafts. The mechanism for the difference in the amount of cholesterol in the DMS vs. the DLS and NAc needs to be unraveled. One possibility is that the cholesterol biosynthesis and/or metabolism is different between these three substriatal regions. For example, it is plausible that cholesterol 24-hydroxylase which is responsible for cholesterol turn over in the CNS (Russell et al., 2009) is differentially expressed in the DMS vs. DLS and NAc.

What could be the consequences of Fyn activation and GluN2B phosphorylation in D1R MSNs in the DMS? Studies in primary and organotypic striatal cultures revealed that activation of NMDA receptors increases the recruitment of the D1Rs into the plasma membrane and specifically into spines (Scott et al., 2002, 2006). It is therefore plausible that in the DMS, D1R-dependent, Fyn-mediated phosphorylation of the GluN2B subunit results in the enhancement of the activity of the channel which in turn leads to further increases in the membrane localization of the D1R, and to further activation of D1R signaling. Furthermore, D1R activation in the striatum was shown to enhance the forward trafficking of the NMDAR subunits and increased dendritic localization and surface expression of GluN2B subunit (Dunah and Standaert, 2001; Hallett et al., 2006). Thus, it is also plausible that the activation of Fyn in DMS D1R MSNs plays a role in the induction of NMDAR-dependent long term potentiation (LTP) which was observed in D1R but not D2R MSNs in the dorsal striatum (Shen et al., 2008).

In summary, our data show that Fyn is compartmentalized differently in the DMS, DLS, and NAc which in turn determines the consequences of D1R activation.

The raw data for this study can be found at https://portal.g-node.org/data/. Readers can access the data by creating an account and searching for “Phamluong.”

## Author contributions

Conceptualization: ED and DR; Methodology: KP, ED, SAS, and SW; Investigation: KP, ED, and SW; Manuscript preparation: DR; Supervision: DR.

### Conflict of interest statement

The authors declare that the research was conducted in the absence of any commercial or financial relationships that could be construed as a potential conflict of interest.
